# “Coffee with Alexa” – Facilitators and Barriers to Adoption of Voice-Activated Virtual Assistants

**DOI:** 10.1093/geroni/igaf122.3874

**Published:** 2025-12-31

**Authors:** Patricia Oh, Susan Wehry, Jennifer Jain, Ruth Dufresne, Katie Keough, Rachel Coleman, Lenard Kaye

**Affiliations:** University of Maine, Bangor, Maine, United States; University of New England, Portland, Maine, United States; University of Maine, Bangor, Maine, United States; Center for Excellence in Public Health, University of New England, Portland, Maine, United States; University of New England, Portland, Maine, United States; University of Maine Center on Aging, Orono, Maine, United States; University of Maine Center on Aging, Orono, Maine, United States

## Abstract

**Background:**

Virtual home assistants (VHAs) have been promoted to support healthy aging and assist people living with early-stage dementia by facilitating daily tasks, providing reminders, and fostering social connections. Adoption is impeded by ageist stereotypes often direct responsibility for installing and monitoring the devices to family care partners. Additional barriers include limited access to affordable internet and insufficient training to use the VHA comfortably.

**Project:**

To evaluate a community-based VHA training and installation program across rural and urban Maine communities that replicated an initiative in an age-friendly suburban community. Twenty-nine volunteers were trained to install VHA in 27 rural communities, each receiving equipment to install VHA in up to 15 homes. Four urban areas participated, each receiving supplies for 25 homes.

**Methods:**

Data collection included volunteer surveys (n = 29), focus groups (n = 24), recipient surveys (n = 201, 68% response rate), and semi-structured interviews (n = 19).

**Results:**

Most volunteers were very comfortable with installation (67%) and had prior VHA experience (59%). Rural best practices included (1) 1:1 custom installation, (2) follow-up, and (3) word-of-mouth recruitment. Urban success depended on (1) collaboration with congregate housing and with an organizational training partner. Among recipients, 72% expressed confidence using their device and 27% customized their VHA routine to meet additional needs after they were installed. Benefits included (1) social connection; (2) independence; (3) fun; and (4) confidence.

**Conclusion:**

These findings challenge ageist assumptions about technology adoption by older adults living with cognitive changes and highlight the potential for community-specific programs with geographically-tailored approaches to distributing VHA.

